# Molecular Update and Evolving Classification of Large B-Cell Lymphoma

**DOI:** 10.3390/cancers13133352

**Published:** 2021-07-03

**Authors:** Arantza Onaindia, Nancy Santiago-Quispe, Erika Iglesias-Martinez, Cristina Romero-Abrio

**Affiliations:** 1 Bioaraba Health Research Institute, Oncohaematology Research Group, 01070 Vitoria-Gasteiz, Spain; 2 Osakidetza Basque Health Service, Araba University Hospital, Pathology Department, 01070 Vitoria-Gasteiz, Spain; NANCY.SANTIAGOQUISPE@osakidetza.eus (N.S.-Q.); ERIKA.IGLESIASMARTINEZ@osakidetza.eus (E.I.-M.); cristina.romeroabrio@osakidetza.eus (C.R.-A.)

**Keywords:** large B-cell lymphoma, next generation sequencing, mutations, pathway activation

## Abstract

**Simple Summary:**

The development of high-throughput technologies in recent years has increased our understanding of the molecular complexity of lymphomas, providing new insights into the pathogenesis of large B-cell neoplasms and identifying different molecular biomarkers with prognostic impact, that lead to the revision of the World Health Organization consensus classification of lymphomas. This review addresses the main histopathological and molecular features of large B-cells lymphomas, providing an overview of the main recent novelties introduced by the last update of the consensus classification.

**Abstract:**

Diffuse large B-cell lymphomas (DLBCLs) are aggressive B-cell neoplasms with considerable clinical, biologic, and pathologic diversity. The application of high throughput technologies to the study of lymphomas has yielded abundant molecular data leading to the identification of distinct molecular identities and novel pathogenetic pathways. In light of this new information, newly refined diagnostic criteria have been established in the fourth edition of the World Health Organization (WHO) consensus classification of lymphomas, which was revised in 2016. This article reviews the histopathological and molecular features of the various aggressive B-cell lymphoma subtypes included in the updated classification.

## 1. Introduction

Diffuse large B-cell lymphoma (DLBCL) is the most common type of lymphoma worldwide. Patients present with a rapidly enlarging tumor mass involving single, multiple, nodal, or extranodal sites A, and approximately 40% of cases are confined to extranodal sites [1,2].

Histologically, these neoplasms are defined as the presence of lymphoma cells larger than the nuclei of histiocytes present in the same section [3,4]. DLBCL encompasses a wide spectrum of neoplasms with morphological and immunohistochemical heterogeneity. Twenty percent of cases are thought to be sufficiently different, and thirteen different variants have been accepted on the last review of the WHO organization, on the basis of distinctive morphological or immunophenotypic findings or distinctive biological issues associated with their diagnoses. However, 80% to 85% of DLBCL cases are not sufficiently distinctive and are therefore designated as not otherwise specified (DLBCL-NOS) [2,5].

A few changes have been introduced in the fourth edition of the WHO classification regarding DLBCL [2]: (1) the “cell of origin” (COO) classification must be included in the pathology report; (2) CD5, MYC, and BCL2 immunohistochemical expression must be assessed as prognostic factors; (3) the provisional entity “B cell lymphoma, unclassifiable, with features intermediate between DLBCL and Burkitt lymphoma” has been replaced by two new categories: “high-grade B-cell lymphoma with MYC and BCL2 and/or BCL6 translocations” and “high-grade B-cell lymphoma, not otherwise specified”.

This review focuses on the molecular update of the different DLBCL variants (Table 1 and Table 2). Viral-driven diffuse large-B cell lymphomas will not be treated in this issue.

## 2. Diffuse Large-B-Cell Lymphoma, Not Otherwise Specified (Dlbcl-Nos)

Diffuse large B-cell lymphoma, not otherwise specified (DLBCL-NOS), accounts for about one-third of all non-Hodgkin’s lymphomas [1]. By definition, DLBCL-NOS is predominantly an aggressive lymphoma that does not belong to a specific subtype and/or variant of DLBCL [2,6]. DLBCL-NOS frequently affects elderly patients with a slight male predominance, although it can also occur in children and young adults [2,6]. Histologically, the involved tissues show diffuse infiltrates of medium- to large-sized neoplastic cells, displaying a centroblastic appearance in 80% of cases. The immunoblastic variant represents 8–10% of cases, and in the rare anaplastic variant (3% of the cases), the tumoral cells are large, bizarre, and pleomorphic [3,7].

Besides this morphologic subdivision, the COO classification has been shown to identify distinct DLBCL prognostic subgroups, with germinal center B-cell (GCB) subtype DLBCL cases (GCB-DLBCLs), representing 40–50% of DLBCL cases, being associated with a significantly better outcome compared to the activated B-cell (ABC) subgroup (ABC-DLBCL) (50–60% of DLBCL cases) [8]. Approximately 10–15% of cases cannot be included in either of these subgroups and remain unclassified [9].

Consistent with their COO, GCB-DLBCL cases harbor hypermutated immunoglobulin genes with ongoing somatic hypermutation and a high level of BCL6 expression, whereas ABC-DLBCL ones show NF-κB- and BCR-signaling-pathway activation [10]. Therefore, ABC-DLBCLs frequently harbor mutations affecting *MYD88* (~20% of cases), *CD79A*/*B* (~20%), *CARD11* (~10%), *MALT1*, *BCL10*, and *TNFAIP3*, responsible for NF-κB pathway activation [11,12]. Additionally, *PRDM1*/*BLIMP1* mutations are also seen in 30% of DLBCL-NOS cases, exclusively in the ABC subtype. In contrast, the GCB-subtype group more often shows mutations involving histone methylation or acetylation genes (*EZH2*, *EP300*, *CREBBP*, *KMT2D*), B-cell homing genes (*GNA13*, *GNAI2*, *SIPR2*), the PI3K signaling pathway, and the JAK-STAT pathway. *EZH2* mutations are exclusive to the GCB-type, being found in approximately 20% of cases. *EZH2* mutations are gain-of-function mutations leading to an increased expression of the EZH2 protein, which is responsible for the methylation of histone H3 at lysine 27, inducing transcriptional repression and gene silencing [13,14,15,16].

The development of high-throughput technologies in recent years has increased our understanding of the molecular complexity of lymphomas beyond COO classification, defining DLBCL genetic signatures, characterizing new DLBCL subsets, and providing new insights into DLBCL pathogenesis. Initially, six genetic subgroups were defined [17,18,19]: MCD, BN2, N1, EZB, ST2, and A53. MCD and N1 cases were mostly ABC-DLBCL cases, with MYD88L265P and *CD79B* characterizing MCD cases and *NOTCH1* mutations being present in the N1 cases. EZB and ST2 DLBCL cases were mostly GCB-DLBCLs, with recurrent *BCL2* translocations and *EZH2* gene mutations in the EZB subtype cases, and mutations in *SGK1* and *TET2* in the ST2 subtype ones [17,18]. Additionally, a worse-prognosis subtype group (A53) was also defined, with cases showing aneuploidy, *TP53* mutations, and deletions [18]. Later on, whole-exome sequencing (WES) data in 304 primary DLBCLs were used by Chapuy et al. [20] to define five DLBCL subtypes (C1 to C5), allowing the distinction of both ABC and GCB-DLBCL cases in two distinct subgroups, with favorable and adverse outcomes. ABC-DLBCLs subtypes clustered in two different groups: a possible marginal zone origin one (C1) with *NOTCH2* mutations, *BCL6* translocation, and a lower risk, and a higher risk group one (C5) with recurrent mutations in *MYD88*, *CD79B*, and *PIM1* [20]. In the same way, two different GCB-DLBCLs subtypes were also defined, including a C4 subgroup with favorable outcomes and a C3 subgroup with poor prognosis. C4 DLBCLs showed mutations affecting RAS/JAK/STAT pathway members (*BRAF*, *STAT3*), BCR/Pi3K signaling intermediates (*RHOA*, *GNA13*, and *SGK1*), KFkB modifiers (*CARD11*, *NFKBIE*, *NFKBIA*) and immune evasion molecules (CD83, CD58, and CD70) [20]. Conversely, C3 DLBCL cases harbored *BCL2* mutations, as well as mutations affecting chromatin modifiers (*KMT2D*, *CREBBP*, and *EZH2*), B-cell transcription factors (MEF2B, IRF8), BCR-and PI3K-signaling modifiers (*TNFSF14*, *HCNV1*, *GNA13*), and *PTEN*-inactivating mutations [20]. Furthermore, a C2 subgroup associated with genomic instability, *TP53* biallelic inactivation, and *CDKN2A* losses was also described [20].

Cytogenetic analysis is also helpful in DLBCL workup. Complex karyotypes are more common in tumors that are clinically aggressive or resistant to therapy, and specific chromosomal aberrations correlate with COO classification. The most frequent translocation (about 30% of cases) implies *BCL6* at the chromosome 3q27 locus [21,22], often juxtaposed with *IGH* on chromosome 14q32 [3], and tends to occur more commonly in the ABC subtype [11,22]. The t(14; 18)(q21; q32)/IGH-BCL2 can be detected in the GCB type [1,7]. Translocations involving *MYC* at 8q24 also occur in 10–15% of DLBCL cases and are often associated with high-grade morphological features and a complex karyotype [23]. A variable proportion of the *MYC* translocations involve the *IG* loci as partners: *IGH*, *IGK*, or *IGL*. Other partners include non-immunoglobulin loci such as *PAX5*, *BCL6*, *BCL11A*, IKZF1 (IKAROS), and BTG1 [24,25].

It is recognized that GEP is not widely available [26,27], but the classic Hans algorithm utilizing antibodies against CD10, BCL6, and MUM-1/IRF4 is routinely used worldwide in COO classification [28] with variable rates of concordance (65–90%) to the GEP classification scheme. With the development of new IHC antibodies, the panel has been expanded in newer iterations, known as the “Choi”, “Tally”, and “Visco-Young” algorithms [29,30,31]. Recently, Yoon et al. [32] also demonstrated that NanoString technology for formalin-fixed, paraffin-embedded tissue may be a robust, reliable method for predicting the outcome, compared to the Hans algorithm.

Therefore, immunohistochemical assessment is mandatory for DLBCL-NOS diagnosis and COO determination, but different IHC marker expressions have been found to have prognostic significance. CD30 expression, frequently seen in the anaplastic variant of DLBCL [33,34], has been shown to be associated with a favorable outcome [34], while CD5-positive cases exhibit features associated with an aggressive clinical course and poorer survival [35]. DLBLC-NOS cases, in which MYC and BCL2 are expressed in >40% and 50% of neoplastic cells respectively, are known as double-expressor lymphomas (DEL). DEL cases are far more common in the ABC subtype and have been shown to have a worse prognosis [26]. PD-L1 and PD-L2 expression has been reported in about 20–25% of DLBCL-NOS cases, correlating with PD-L1/L2 amplification at chromosome 9p24.1 and response to PD1 inhibitors [36]. P53 expression is seen in 20–60% of cases, suggesting an upregulation of wildtype *TP53* in some non-TP53 mutated cases [37].

Morphologic DLBCL variants have specific molecular features: the centroblastic variant is more frequently found in the GCB subtype, while immunoblastic lymphomas are more often found in the ABC subtype [38]. Additionally, immunoblastic lymphomas have been found to frequently harbor *MYC* translocations [39], which confer a worse prognosis. Li et al. [40] recently indicated the distinctiveness of the anaplastic variant based on genetic analyses. They found a higher incidence of p53 positivity (in 80% of the cases) and expression of both MYC and BCL2 (43%) in association with an aggressive clinical course.

## 3. High Grade B-Cell Lymphomas (Hgbl)

Morphologic distinction between Burkitt lymphoma (BL) and DLBCL is a historical diagnostic issue. However, gene-expression profiling studies have shown that BL cases exhibit a specific molecular signature, while other cases within the morphological spectrum of DLBCL and aggressive B-cell lymphomas have an intermediate molecular profile. Therefore, the so-called “grey zone lymphomas” category has been removed and replaced with two new categories: “HGBL with MYC and BCL2 and/or BCL6 rearrangements”, or “HGBL-DH/TH”, and “HGBL not otherwise specified” (HGBL, NOS) [2]. 

### 3.1. High Grade B-Cell Lymphoma with Myc and Bcl2 And/Or Bcl6 Rearrangement (Hgbl with Myc, Bcl2, And/Or Bcl6; Hgbl-Dh/Th)

DH and TH lymphomas are aggressive mature B-cell lymphomas that represents 1% to 12% of DLBCL cases [41]. Many cases morphologically resemble BL, but exhibit greater cytologic pleomorphism or an aberrant immunophenotype with a lower Ki-67 proliferation index or strong BCL2 expression, which are not characteristic of BL. By definition, HGBL DH/TH harbor rearrangements of *MYC* and *BCL2* or *BCL6* (double-hit lymphoma, DHL) or both *BCL2* and *BCL6* (triple-hit lymphoma; THL), detected by conventional cytogenetics or fluorescence in situ hybridization (FISH) [42].

In terms of frequency of occurrence, all three entities exhibit similar demographic, clinical, and morphological features [43,44]. The median age of affected patients is in their 6th decade with a slight male predominance, and most patients have an advanced stage disease (III/IV), with two or more sites of extranodal involvement at diagnosis [45]. Virtually any site can be involved, but bone marrow, peripheral blood, pleural effusions, central nervous system (CNS), and the gastrointestinal tract are frequently affected [45,46,47]. DH/THLs are generally refractory to standard chemotherapy regimens, and they carry a poor prognosis [48].

*MYC*/*BCL2* DHL cases are the most frequent (60–65%), followed by MYC/BCL2/BCL6 THL cases (15–20%) and *MYC*/*BCL6* DHL cases (10–15%) [49]. DH/THLs include cases with classical morphological DLBCL features (centroblastic, immunoblastic, and other rare variants) as well as cases with a blastoid appearance or cytologic features intermediate between BL and DLBCL [2]. Based on the COO classification, almost all DH/THL cases harboring *MYC*/*BCL2* translocations have a GCB phenotype, whereas DHL cases with *BCL6* rearrangement have been observed in both the GCB and non-GCB subtypes [41,42]. Most cases do not express TdT, but a few DHLs or THLs show unequivocal TdT expression. In the updated WHO classification scheme, it is recommended that such cases be diagnosed as B-lymphoblastic leukemia/lymphoma (B-LBL).

*MYC*/*BCL2* DHLs show CD10, BCL2, and BCL6 expression in more than 90% of cases [50], with a ki-67 proliferation rate higher than 80% of neoplastic cells. EBER expression is almost always absent [47]. Most cases show bright CD38 expression with dim CD45 by flow cytometry in about one-third of cases [51]. Conventional cytogenetic studies of *MYC*/*BCL2* DHLs have shown that approximately half of the translocation partners of MYC are the immunoglobulin genes. Immunoglobulin heavy chain (*IGH* at 14q32) is the most common YC translocation partner, followed by lambda light chain (*IGL* at 22q11) and kappa light chain (*IGK* at 2p12) in *MYC*/*BCL2* DHL. In the other half of MYC/*BCL2* DHL, *MYC* partners with non-immunoglobulin gene chromosomal loci, including 1p36, 3p25, 3q27(BCL6), 4p13, 5q13, 9p13 (PAX5), 12p11, and 13q31 [52,53].

Controversy exists regarding the role of the *MYC* partner (*IG* versus non-*IG*) in outcome, with contradictory results. McPhail et al. [54] investigated 52 cases in which the *MYC* rearrangement partner was an *IG* gene, and 35 cases in which the *MYC* rearrangement partner was a non-*IG* gene, observing no association between the *MYC* rearrangement partner and overall survival. Unlike the previous study, Rosenwald and colleagues [55] reported a negative prognostic impact in the *MYC-IG* group by initial FISH assay of *MYC* status. They initially tested DLBCL cases with break-apart probes, followed by testing in the rearranged cases with *MYC*/*IG* heavy chain fusion, *MYC*-*IG* kappa, and *MYC*-*IG* light fusion probes [55]. Thus, screening for *MYC* rearrangement with break-apart probes and further testing with *MYC –IGH*, *IGK* and *IGL* dual fusion probes may improve sensitivity, although with unknown prognostic impact [55,56].

Genomic complexity is high in *MYC*/*BCL2* DHLs, with frequent gains of chromosomes 7, 8, 11, 12, 18, 20, and X, and losses of 3q27–29, 6q, and 15q26 [57]. The mutation profile of *MYC*/*BCL2* DHLs is intermediate between BL and shows mutations affecting *ID3*, *CCND3*, *MYC,* and *TCF3*, and DLBCL of the GCB type, which harbors mutations in *BCL2*, *EZH2*, *CREBBP*, *EP300*, *MEF2B*, and *SGK1* [58]. Evrard et al. [59] reported *CREBB* as the most common in *MYC*/*BCL2* DHLs, followed by mutations affecting *BCL2*, *KMT2D*, *MYC*, *EZH2*, *IGLL5*, *FOX01*, and *SOCS1*. Momose and colleagues [58] also found mutations affecting *ID3* and *CCND3*, similar to BL. *TP53* mutations are also common in this subset of DH/THL, whereas they are not found in *MYC*/*BCL6* rearranged lymphomas [23].

*MYC*/*BCL6* DHLs show similar frequency of immunoglobulin and non-immunoglobulin *MYC* translocation partners as *MYC*/*BCL2* DHL cases. MUM-1/IRF4 and BCL2 expression are variable, with less frequent CD10 expression than in *MYC*/*BCL2* DHLs [49]. Compared to *MYC*/*BCL2* DHLs, bright CD38 expression is present in a lower number of cases (42% of *MYC*/*BCL6* DHL cases compared to 60% of *MYC*/*BCL2* DHLs [49]), and *BCL2*, *TP53*, and *EZH2* mutations are rare in MYC/BCL6 DHL [60].

Similar to *MYC*/*BCL2* DHLs, immunoglobulin and non-immunoglobulin MYC translocation partners are observed almost equally [44] in THL cases. CD10 expression is more frequently found [44]. The mutation profile is similar to other DHL cases, with mutations in *BCL2*, *CCND3*, *CREBBP*, *EZH2*, *ID3*, *KMT2D*, *MYC*, *FOXO1*, and *SOCS1* [58,59].

### 3.2. High Grade B-Cell Lymphoma, Not Otherwise Specified (Hgbcl, Nos)

High-grade B-cell lymphoma, not otherwise specified (HGBCL, NOS) comprises a heterogeneous group of aggressive mature B-cell neoplasms that do not meet the criteria for DLBCL, NOS, or BL and do not harbor double- or triple-hit genetic abnormalities [2]. On morphological grounds, two different variants have been described, including cases with features intermediate between DLBCL and BL (BCL-U) and blastoid morphology, without a genetic double- or triple-hit [2]. Cases with a BCL-U morphology are composed of a diffuse proliferation of intermediate-sized cells with a starry-sky appearance and strong mitotic activity, but that do not fit the classical BL definition either due to BCL2 diffuse immunohistochemistry expression or karyotypic abnormalities [23]. Cases with blastoid morphology resemble lymphoblasts and overlap with blastoid mantle-cell lymphomas, showing intermediate-sized cells with blastoid chromatin and a starry-sky appearance [42]. Sixty percent of cases with blastoid morphology belong to the DH/TH category, after FISH testing [42,61,62].

Several approaches have been considered for the selection of cases for further FISH testing, including KI-67 proliferation index assessment [61], MYC and BCL2 double immunohistochemical expression [41], and a COO phenotype study following the Hans algorithm [41], but none of them have been shown to be sensitive and specific enough. Therefore, FISH testing for MYC translocation is recommended in all cases with DLBCL, BCL-U, and blastoid morphology [42].

At the molecular cytogenetic level, HGBCL, NOS cases may harbor isolated *MYC*, *BCL2*, or *BCL6* breakpoints. *MYC* is the most frequent sole rearrangement present in these neoplasms and is associated with a poor prognosis, with similar progression-free survival rate (PFS) and overall survival rate (OS) as DHL [63]. *BCL2* and *BCL6* can also be rearranged simultaneously without *MYC* rearrangement, and extra copies or the amplification of *MYC*, *BCL2*, or *BCL6* [23,64] can be observed.

### 3.3. Burkitt Lymphoma (BL)

BL is a highly aggressive B cell NHL, representing less than 5% of adult lymphoma cases and 40% of all childhood NHL [2]. BL derives from mature germinal or post-germinal center B cells and is divided into three clinical entities, all of which present rearrangements of the *MYC* oncogene contributing to the overexpression of C-MYC [65]. BL may present as a leukemic form corresponding to Burkitt cell acute lymphoblastic leukemia (L3ALL), according to the revised 2016 World Health Organization (WHO) classification of hematologic malignancies [42].

Three different clinical variants of BL have been identified, sharing similar morphology, immunophenotype, and genetic features: endemic, sporadic, and immunodeficiency-related. The “endemic” variant (eBL) occurs in equatorial Africa, presenting with head and neck jaw affection and Epstein–Barr virus (EBV) infection in almost all cases [66,67]. EBL exhibits an improved outcome, with survival rates higher than 90% even in patients with central nervous system (CNS) involvement [68]. Concurrent infection with *Plasmodium falciparum* and EBV are regarded as the main risk factor for the development of EBL [69]. However, EBV is associated at different frequencies with sporadic BL, depending on the geographic regions [70], varying from 10 to 25% [70,71,72]. A subpopulation in southeastern Brazil was found to show an intermediate frequency of EBV infection between endemic and sporadic subtypes (60%) [72], and different studies have demonstrated that the frequency of EBV infection in HIV-associated BL cases ranges from 25% to 50% [70]. The role of EBV in BL is still unclear, and it has been suggested that EBV-positive and -negative cases might arise from different cells of origin [73].

Histologically, BL is characterized by an extremely high proliferation index (almost 100%) with a high turnover associated with a high apoptosis rate and a starry-sky appearance. The cells are intermediate in size, with a deeply basophilic cytoplasm containing small lipid vacuoles. The nuclear chromatin is granular and contains small nucleoli with frequent mitoses. Some nuclear pleomorphism is observed due to some plasmacytoid and atypical variants. BL is derived from a germinal center derived lymphoma, exhibiting BCL6, CD10, CD19, CD20, CD22, and CD79a expression and lacking CD5, BCL2, TdT, and, typically, CD23.

Virtually all cases of BL harbor a *MYC* translocation that causes a constitutive overexpression of the MYC oncogene. The t(8; 14) (q24; q32) is the most common chromosomal translocation, occurring in 80% of BL. It concerns the long arm of chromosome 8, the site of the MYC oncogene (8q24), and the immunoglobulin heavy chain (IGH) locus on chromosome 14. The t(2; 8)(q12;24) and t(8; 22) (q24;11), which correspond to the kappa (IGK) and lambda light chain (IGL) loci on chromosomes 2 and 22, are found in 15% and 5% of patients, respectively [74]. It is well-established that MYC translocation is not a sufficient genetic event to cause BL.

NGS techniques have provided a comprehensive analysis of the landscape of additional genetic events that contribute to BL lymphomagenesis. Burkitt lymphoma survival is dependent on “tonic” BCR signaling, which activates the *PI3K* pathway in BL cells, and the consequent signaling cascade promotes proliferation [10]. Approximately 70% of BL cases show constitutively active *PI3K* signaling [75]. This aberrant signaling results from somatic mutations in *TCF3* or its negative regulator *ID3*, which encodes a protein that blocks *TCF3* activity. Thirty-eight percent of sporadic BL cases harbor a mutation in CCDN3, which is activated by *TCF3* and encodes cyclin D3, which promotes cell cycle progression [74]. Mutations of the transcription factor *TCF3* or its negative regulator *ID3* (DNA binding protein family) were detected in 70%, 67%, and 40% of patients with sporadic, endemic, and HIV-associated BL respectively [2,10]. Recent mutational cartography efforts in Burkitt lymphoma identified additional recurrent mutations in *GNA13*, *RET*, *PIK3R1*, *DDX3X*, *FBXO11*, and the *SWI*/*SNF* genes *ARID1A* and *SMARCA4* [76].

Panea et al. [77] applied whole-genome sequencing (WSG) and transcriptome sequencing to comprehensively investigate the genome basis of all three subtypes of BL. They found that *BCL7A* and *BCL6* genetic events were enriched in the endemic subtype, whereas *DNMT1*, *SNTB2*, and *CTCF* mutations occurred more frequently in HIV and sporadic patients. *SNTB2* mutations were associated with EBV-negative patients, and *IGLL5* and *BACH2* mutations were associated with EBV-positive patients [77]. Targeting the PI3K pathway, MYC and TCF3 expression, and cell cycle kinases are among many therapeutic options to be tested in future clinical trials [78,79].

About 40% of BL acquire *TP53* mutations evading MYC-induced stress signals [80]. Targeting *MDM4* to alleviate the degradation of p53 can be exploited therapeutically across BL and other cancers with wild-type p53 harboring 1q gain, the most frequent copy number alteration in cancer [81]. Artesunate could also inhibit the proliferation and induce ferroptosis of BL cells in vivo, causing a significant endoplasmic reticulum stress response in the tumor cells.

### 3.4. Burkitt-Like Lymphoma with 11q Aberration (Bll, 11q)

Burkitt-like lymphoma with 11q aberration (BLL, 11q) is a rare diagnostic entity newly recognized as a provisional entity in the revised 4th (2017) edition of the WHO classification as a group of lymphomas resembling BL morphologically, phenotypically, and by gene and microRNA expression profiling, but lacking the characteristic *MYC* rearrangement seen in BL [2].

Significant morphological overlap exists between BL, DLBCL, and HGLBCL. BL11q cases show a diffuse proliferation of intermediate cells, with small nucleoli and frequent mitotic features and apoptotic bodies [82,83,84,85] imparting a starry-sky pattern similar to BL and HGLBCL, while increased pleomorphism and a lack of macrophages can assist with differential diagnosis with DLBCL [83,86,87]. However, weak MYC expression and increased LMO2 expression may be useful markers in the differential diagnosis between these entities [16,88].

NGS and DNA copy number analysis studies [14,16,75,76,89] have provided a list of genomic alterations distinguishing BL from DLBCL. Recently, Gonzalez-Farre et al. [83] identified genetic events suggesting BLL-11q is closely related to HGLBCL and DLBCL, rather than BL. This study [83] demonstrated that BLL-11q lacks *ID3*, *TCF3*, and *CCND3* mutations, which are frequently found in BL, and shows, by contrast, frequent gains of 12q12-q21.1 and losses of 6q12.1-q21, which can be seen in 15% of DLBCL and *IRF4*-translocated lymphomas [86]. BLL-11q also shows potential specific driver mutations involving *BTG2*, *DDX3X*, *ETS1*, *EP300*, and *GNA13*, indicating that BLL-11q is a distinct lymphoma from BL at the molecular level [90].

However, 11q-gain/loss is not specific to this subgroup of lymphomas and can be seen in MYC-positive BL and MYC-positive (HGBL-NOS). This finding indicates that 11q aberration can be a primary or a secondary genetic change in the development of aggressive B-cell non-Hodgkin lymphoma (B-NHL) [84].

## 4. T-Cell/Histiocyte-Rich Large B Cell Lymphoma

T cell/histiocyte-rich large B cell lymphoma (THRLBCL) is an uncommon and aggressive variant of DLBCL [2], presenting with advanced stage disease and frequent spleen and liver involvement cells [2,91] in middle-age patients.

THRLBCL shows scattered large blasts with Hodgkin’s Reed–Sternberg cell-like nuclear features, with a popcorn-like and centroblast-like morphology, in a polymorphic background rich in epithelioid histiocytes and reactive T-cells. Neoplastic cells show pan B-cell markers (CD19, CD20, and CD79a), with BCL-6 germinal center marker expression, and PD-L1 and c-MYC positivity [2]. Background-reactive T cells show a predominance of T CD4+ cells, with a few IgD-positive B naïve cells [2,91]. Follicular dendritic cell meshwork is absent, and PD-1 positive T cells adopting a rosette configuration around tumor cells are infrequently observed [92,93].

Histological, clinical, and genetic overlap exists between NLPHL and THRLBCL, suggesting both entities may represent a biological continuum [91,94,95]. Immunoglobulin gene region studies [96,97,98] have demonstrated shared clonality between NLPHL and THRLBCL, indicating that 2% to 17% of THRLBCL come from the transformation of NLPHL [99,100]. Risk factors for transformation are not known, although variant growth NLPHL patterns (C-F) [101] with extracellular and diffuse LP patterns in a T-cell rich nodular background or a diffuse moth-eaten B-cell pattern are associated with a more advanced disease presentation [102]. Spleen involvement at time of NLPHL diagnosis might represent a risk factor for transformation [99].

A high level of genomic similarity between NLPHL and THRLBCL has also been demonstrated [94,95,103]. Gene-expression profiling (GEP) studies showed that NLPHL and THRLBCL cluster together [95] with no consistently differentially expressed genes between them [94,95,103]. *UBD*/*FAT10* and *BAT3*/*BAG6*, both interacting with the tumor suppressor gene *TP53* were found to be expressed in both entities [94,102]. Comparative genomic hybridization (CGH) studies also revealed similarities between both lymphomas, with common gains of 2p16.1 and losses of 2p11.2 and 9p11.2 [102,104]. The commonly gained region 2p16.1 includes *PEX13*, *REL*, *BCL11A*, *PAPOLG*, *KIAA1841*, *PUS10*, and *LINC085* genes, while *FABP1*, *EIF2AK3*, *KRCC1*, *THNSL2*, *SMYD1*, *FOXI3*, *SPATA1A7*, and *AQP7P1* are frequently lost. Frequent gains on the *REL* locus on 2p16.1, with increased REL expression have also been identified [103]. PAX5/IGH rearrangements have been reported [105], while BCL6 translocations have not been described yet in THRLBCL, as opposed to NLPHL [106]. Strong KFkB activity [95] is common in both NLPHL and THRLBCL.

Ultra-deep targeted resequencing of THRLBCL cases identified recurrent mutations with enrichment of somatic hypermutations in *JUNB*, *DUSP2*, *SGK1*, and *SOC1* [107,108]. Tian et al. [109,110] also demonstrated EZH2 overexpression in THRLBCL, which was correlated with proliferation rate and intracellular-signaling-cascade-associated molecules p-ERK1/2, MYC, and p-STAT3.

Despite histological and molecular similarities, behavioral differences might be related to the tumor microenvironment. In the typical patterns, LP cells in NLPHL are surrounded by resetting PD1-positive T cells, with frequent CD4+ CD8+ double-positive T cells [111] and a high number of CD69+ T-cells, which are supposed to have an immunosuppressive effect by TGF-β production [112]. By contrast, THRLBCL is characterized by a decreased proportion of CD4+ cells, compared to NLPHL, and a dominant content of macrophages and dendritic cells, which have been show to create a tumor tolerogenic environment [113]. THRLBCL microenvironment’s signature, however, is hallmarked by the upregulation of CCL8, interferon-ϒ, indoleamin 2,3 dioxygenase, VSIG4, and Toll-like receptors, which are responsible for the activation of T-cells, innate immunity, and tolerogenic immune response in THRLBCL [113]. Recently, Griffin et al. [114] have reported that *PD-L1*/*PD-L2* copy gains in neoplastic cells in up to 64% of THRLBCL cases, with an associated increase in PD-L1 expression, and clinical responses to PD-1 blockade in 60% of patients with THRLBCL.

## 5. Diffuse Large B-Cell Lymphoma at Specific Sites

### 5.1. Large B-Cell Lymphoma with Irf4 Rearrangement

Large B-cell lymphoma with *IRF4* rearrangement (LBCL-IRF4) is a new provisional entity recently included in the WHO classification [2,115], which was initially described in 2011 by Salaverria et al. [116]. LBCL-IRF4 is a very infrequent neoplasm accounting for 0.05% of diffuse large B-cell lymphomas [2]; it primarily affects children and young adults with a median age of 12 years [2,116] and is characterized by a strong expression of IRF4/MUM1 and frequent *IRF4* rearrangement [2].

LBCL-IRF4 usually presents with localized disease involving predominantly Waldeyer’s ring or lymph nodes of the head and neck region [2,116,117]. Only a few cases are reported involving sites below the diaphragm (abdominal lymph nodes and gastrointestinal tract [116,118]). Patients with LBCL-IRF4 have a favorable prognosis after treatment with immunochemotherapy with or without radiation [116,119].

Morphologically, most LBCL-IRF4 cases have at least a partial follicular growth pattern resembling FL grade 3B, although diffuse areas [2,119], with medium to large neoplastic cells that can show blastoid appearance and high-grade features are consistent with DLBCL. Follicular areas are characterized by large, tightly packed follicles without polarization and indistinct or absent mantle zones. A starry-sky pattern is rare and mitotic figures are present in moderate number [2].

Immunophenotypically, tumor cells express B-cell markers (CD20, CD79a, PAX5) with a strong expression of IRF4/MUM1 and BCL6 [2]. CD10 and BCL2 are shown in approximately 50% of cases [120], although it lacks t(14;18). The proliferation rate is commonly high, with no evidence of polarization in the neoplastic follicles [2,121]. Rare cases are CD5-positive, especially CD10-negative ones, though a double expression of CD5 and CD10 has been reported [122]. Some features may prompt the pathologist to suspect LBCL-IRF4: the young age of the patient, the peculiar location and histopathological pattern with typical centroblasts in follicular and diffuse growth, and the characteristic immunoprofile including the positivity of CD10, BCL6, and IRF4/MUM1 [123].

Most cases of LBCL-IRF4 are found to have a germinal center B-cell (GCB) origin, with a unique gene-expression signature [116,120]. The presence of IRF4 rearrangements identified by FISH studies, together with *BCL6* rearrangements and a uniform lack of *BCL2* and *MYC* rearrangements, are the defining features of LBCL-IRF4 [116,120,124]. Some cases may additionally exhibit *IRF4*/*IGH* fusions but are negative for IRF4 rearrangement using commercially available break-apart probes [125]. The involvement of light chain genes has been rarely reported [116]. Salaverria et al. [126] showed complex genomic changes using comparative genomic hybridization techniques, such as gains of 7q32.1-qter, 11q22.3-qter, and Xq28 and losses of 6q13–16.1, 15q14–22.3, 17p, and *TP53*. However, these molecular alterations were not predictors of clinical behavior. In contrast, Ramis-Zaldivar et al. [127] suggested that the underlying mutational profile may influence the morphological feature of tumors. They found that these tumors have additional mutations in NF-kB-related genes (*CARD11*, *CD79B*, and *MYD88*) and overexpression of genes of the KFkB pathway. 

### 5.2. Primary Mediastinal (Thymic) Large B-Cell Lymphoma

Primary mediastinal large B-cell lymphoma (PMBL) is a distinctive clinicopathological entity with specific molecular features, accounting for 6–10% of all DLBCL, which is thought to arise from B-cells in the thymus [2,128]. PMBL affects predominantly women with a median age of 35–37 years, presenting with an enlarging often >10 cm in diameter (bulky) mediastinal mass [128] and superior vena cava syndrome. Most patients present with low stage disease (I/II), with frequent supraclavicular lymphadenopathy [129]. Histologically, PMBL is characterized by a diffuse infiltration by neoplastic cells with round or pleomorphic nuclei, pale and clear cytoplasms, and Reed–Sternberg-like cells, admixed in a sclerotic background [130].

Neoplastic cells are positive for pan-B-cell antigens, with frequent OCT-2 and BOB.1 expression, BCL6 and MUM1 expression in 95% of cases, and C-MYC and BCL2 in 65%. CD23 is observed in 70–95%, and CD30 is seen in 80% of cases [131]. PMBL is characterized by NF-kB pathway activation, with c-REL and TRAF1 expression in 65 and 62% of cases, respectively [131], as well as MAL and CD200 expression in 72 and 81%, respectively [131].

PMBL is genetically defined by the activation of different pathways, including the NF-kB, cell-cycle dysregulation, apoptosis, JAK-STAT, and immune-evasion pathways. Amplification of *REL* (75%) and *BCL11A* (50%), as well as deletions of *NFKBIE* (20%) and bi-allelic mutations of *TNFAIP3* encoding (60%), are alterations frequently seen in PMBL dysregulating the NF-kB pathway [132,133]. Major genetic alterations involving apoptosis and cell-cycle regulation include *GNA13* (50%), *BCL6* (50%), and *XPO1* (35%) [134]. Gains and amplifications of JAK2, PD-L1, and PD-L2, located on chromosome 9p24.1, are seen in up to 70% of PMBL cases [135], inducing PDL-1 and STAT3 overexpression. *STAT6*, *SOCS1*, *PTPN1*, and *IL4R* mutations have also been described [136,137]. Different genetic alterations contributing to immune system evasion have been reported, such as those affecting *CIITA*, *CD58*, *B2*, *PD-L1*, and PD-L2 [137,138].

### 5.3. Primary Cutaneous Diffuse Large B-Cell Lymphoma, Leg Type

Primary cutaneous diffuse large B-cell lymphoma, leg type (PCDLBCL-LT) is a rare but aggressive lymphoma, presenting with generally rapidly growing solitary or multiple ulcerated skin masses, typically on the lower legs [2], affecting elderly female patients. PCDLBCL-LT can also arise on the trunk, head–neck, and upper arms in 15% to 20% of cases [139,140]. Extracutaneous spreading has been reported with frequent lymph node, bone marrow, and central nervous system involvement [2,141,142,143,144].

Previous studies revealed an apparent worse prognosis for lesions located on the leg and for the presence of multiple skin lesions at diagnosis, with a 30–50% 5-year overall survival rate [139,140,145]. However, recent studies have reported a significantly better clinical outcome for patients when rituximab is added to a multiagent chemotherapy regimen [145,146].

Histologically, the dermis and/or subcutaneous tissue are infiltrated by diffuse sheets of medium to large centroblasts and immunoblasts [1,131], with little stromal reaction, few infiltrating small non-neoplastic lymphocytes, and frequent mitoses. The epidermis is uninvolved in most cases [141]. Pan B-cell markers are typically positive, with BCL2, BCL6, IRF4/MUM1, FOXP1, and IgM expression in most cases (>85%) [141,147,148,149,150] and a lack of CD10 immunoreactivity [139,148,150]. Up to 80% of cases exhibit c-MYC positivity, and approximately two-thirds of cases have a double-expressor phenotype [151]. Immunohistochemical expression of p63 is also variable but seen in most cases [152]. BCL-2 overexpression is associated with a chromosomal amplification of the *BCL-2* gene in some cases, but t(14;18) is not usually found [153,154]. PD-L1 and PD-L2 can be expressed on a subset of cells [155].

Previous studies [156,157] have suggested that the PCDLBCL-LT mutational profile is closer to that of primary central nervous system lymphoma rather than the DLBCL subtypes. PCDLBCL-LT shows an activated B-cell (ABC) subtype genotype [158] with frequent monoclonal *Ig* rearrangements, somatic hypermutations, and translocations involving *BCL6*, *MYC*, and *IGH* [2,159].

Several gene-mutation analyses have shown mutations affecting *MYD88* (up to 75% of PCDLBCL-LT cases), *TNFAIP3* (~40%), and B-cell receptor pathway genes (*CD79A*, *CD79B*, *CARD11*), which are responsible for the constitutive activation of the NF-ƙβ pathway and ABC genotype and correlate with poorer outcome [135,160]. Assessment of *MYD88* mutational status has been proposed as a useful diagnostic marker in PCLBCL-LT [160,161].

*MYC* translocations have been associated with a higher risk of disease progression and shortened overall survival [162]. Amplification of chromosome 18q21 region, where the *BCL2* and *MALT1* genes are located [154,156], loss of the *CDKN2A* and *CDKN2B* gene loci on chromosome 9p21.3, and 6q deletions with losses of the *BLIMP1* gene [139,154,156] are recurrent cytogenetic events in PCDLBCL-LT.

Recently, new genomic changes, such as *NFKBIE* mutations and *IGH-IRF8* translocation, have been found in a patient who progressed after ibrutinib treatment, and PD-L1/PD-L2 translocations have been reported in up to 40% of PCDLBCL-LT cases [156,163]. Mitteldorf et al. [164] suggested that PD-L1+ tumor microenvironment (TME) cells induce apoptosis in PD-L1+ tumor-infiltrating lymphocytes, and, therefore, direct inhibition of TME cells, particularly myeloid-derived suppressor cells, could be a promising treatment option.

### 5.4. Primary Diffuse Large B-Cell Lymphoma of the Central Nervous System

Primary diffuse large B-cell lymphoma of the CNS (CNS-DLBCL) is a rare and aggressive subtype of DLBCL confined to the CNS (brain, spine, leptomeninges, or eyes) [2], accounting for <3% of all newly diagnosed intracranial neoplasms and <1% of all non-Hodgkin lymphomas [165,166,167].

CNS-DLBCL shows a diffuse proliferation of large atypical lymphoid cells with a perivascular growth pattern and large areas of geographical necrosis, with immunoblastic, centroblastic, or anaplastic features, and with a prominent astrocytic and microglial activation [2,168]. The neoplastic cells express pan B-cell markers (PAX5, CD19, CD20, CD79a). Most CNS-DLBCLs exhibit a non-germinal B-cell immunophenotype (non-GCB) according to the Hans classification, with BCL6 and MUM1 expression in 60–80% of cases and 90%, respectively [169,170].

*MYC*, *BCL2*, and *BCL6* translocations have been related to an aggressive clinical course and response to conventional chemotherapies [171,172,173,174]. Lower frequencies of these rearrangements in CNS-DLBCL compared to systemic DLBCL suggest that brain environment may play an important role in its pathogenesis [171,172,173,174].

On the other hand, the expression of histone methyltransferase EZH2 has been found in 20% of germinal center B-cell-like (GCB) DLBCLs and plays an important role in its tumorigenesis [175,176]. Guo et al. [177] revealed that EZH2 is strongly expressed in primary CNS-DLBCL, with no evidence of Y641 mutation [177,178]. 

Recurrent copy number abnormalities have also been described, including gains of genetic material affecting the 18q21.33–23 regions, which includes the *BCL2* and *MALT1* genes, as well as the 10q23.21 region in chromosome 12. Deletions of 6p21, 6q, and 9p21.3 have also been described [2,179,180].

Advances in NGS have enabled the effectiveness and comprehensive analysis of the molecular composition and function of CNS-DLBCL. In some cases, these mutations affect potential therapeutic targets and provide new opportunities for personalized therapies. Mutations have been categorized into eight cellular pathways: NF-kB (*PIM1*, *MYD88*, *IRF4*, *CARD11*), B-cell receptor (*CD79B*, *CD79A*, *ITPKB*), epigenetic modulation (*KMT2D*, *CREBBP*, *MEF2B*), JAK-STAT (*CCND1*, *SOCS1*, *STAT6*, *STAT3*), NOTCH1/2, immune related (*CD58*, *CIITA*), PI3K-AKT, and apoptotic regulation (*GNA13*, *MYC*, *RHOA*, *BCL2*) [181,182]. The most commonly mutated genes include *MYD88,* which is found in more than half of the cases [183], *PIM1*, *CD79B*, and *CDKN2A* [181,184]. Lionakis et al. [185] showed that the *MYD88* and *CD79B* pathways targeted with ibrutinib, a Bruton tyrosine kinase (BTK) inhibitor, appears to enhance the efficacy of chemotherapy. Balint et al. [186] also pointed out the potential role of mutations affecting the *PTEN* and *SMO* genes in CNS-DLBCL pathogenesis, as well as their correlation with inferior overall survival. Zheng et al. [187] showed that 37% of their cases had methylation in the promoter in exon 1 of the *MGMT* gene. Thus, targeting the MGMT protein could represent a potential drug target for novel agents in the treatment of this aggressive lymphoma. *TP53* mutation status in CNS-DLBCL remains unclear. A few studies revealed incidences <10% [188,189], while, paradoxically, other investigations show higher rates (29–79%) of p53 protein expression by immunohistochemistry [190,191,192,193]. 

Rare CNS-DLBCL cases occur in an immunosuppression context with frequent EBV infection and association with a dismal outcome. EBV-positive cases lack the frequent mutations found in the *MYD88*, *CD79B*, and *PIM1* genes in the EBV-negative cases, and rarely showed copy number loss in HLA class I/II and antigen-presenting/-processing genes [194]. These results suggested a different pathobiological origin between EBV-positive and -negative CNS-DLBCLs [194].

CNS-DLBCL is an entity distinct from systemic DLBCL. Moreover, because it resides in an immune-privileged site, CNS-DLBCL may have a different immune microenvironment. Kim et al. [174] investigated PD-L1 immunostaining expression on tumor and non-tumoral cells, as well as the presence of PD-1+ and CD8+ tumor-infiltrating lymphocytes (TILs). PD-L1 was either frequently expressed in tumor cells and the microenvironment or further positively correlated with increased TILs. Frequent copy number gains at chromosome 9p24.1, which includes the programmed death ligand 1 and 2 (PD-L1/PD-L2) locus, have been reported [179,195,196]. Up to 30% of patients showed PD-L1 expression by the neoplastic cells in the study performed by Nayyar et al. [182], despite not identifying copy gains of *PD-L1*/*PD-L2.* As we mentioned previously, tumoral PD-L1 expression has been described to be associated with a worse prognosis, and a large number of CD8+ or PD-1+ TILs have been associated with a favorable outcome. According to Furuse et al. [197], PD-L1 expression on peritumoral macrophages is correlated with longer overall survival.

These recent molecular insights into the underlying genomic alterations are relevant to the CNS-DLBCL clinical trials for refractory or relapsed cases that are currently assessing the efficacy of small molecules. Immune checkpoint inhibitors, rapamycin or immunomodulatory drugs (IMiDs), targeting the PI3K/mammalian (mTOR) pathway and BCR/TLR pathways, respectively, show promising clinical responses [198,199].

## 6. Large B Cell Lymphomas with Plasmacytic Differentiation

### 6.1. ALK-Positive Large B-Cell Lymphoma

ALK-positive large B-cell lymphoma (ALK+ DLBCL) is a newly recognized entity by the fourth edition of the WHO [2] because of its distinct characteristics and clinicopathological significance [2]. ALK+ DLBCL is a rare and aggressive form of B-cell lymphoma exhibiting plasmablastic differentiation, which was initially described in 1997 by Delson and colleagues [200] as an immunoblastic or plasmablastic large cell lymphoma with constant ALK immunohistochemical expression. Histologically, ALK+ DLBCL shows a sinusoidal growth pattern, plasmacytic and T-cell lineage markers with CD30 and single light-chain cytoplasmic immunoglobulin A (IgA) expression, and a lack of CD20 and CD79α markers.

As mentioned above, ALK immunohistochemical expression is a constant finding in ALK + DLBCK, showing different patterns according to the underlying gene rearrangement.

Firstly, molecular and protein analyses failed to reveal an ALK gene rearrangement, but later studies demonstrated that most ALK+ DLBCL harbor the t(2;17)(p23;q23) translocation, which results in the fusion of the ALK gene at chromosome band 2p23 and the clathrin gene (CLTC) at 17q2.clathrin (CLTC), in 85–90% of the cases [201,202], and more rarely, the t(2;5)(p23;q35), which results in the nucleophosmin (NPM)-ALK fusion gene, which characterizes up to 75% of the anaplastic large T cell lymphomas (ALCL) [203,204]. Stachursky et al. [205] also identified a cryptic insertion of the *ALK* gene into chromosome 4 at band 4q22–24, and additional *ALK* partner genes have been identified, including *SEC31A*, *SQSTM1*, *RANBP2*, and *IGL* [203,204,206,207].

*NPM-ALK*, *CLTC-ALK,* and *SQSTM1-ALK* translocations lead to ALK and STAT3 phosphorylation [207] with downstream oncogenic activation of the transcription factor STAT3, which enhances lymphoma cell proliferation and growth. Consequently, STAT3 inhibition provides a possible therapeutic target al.so for ALK + DLBCL [207].

### 6.2. Intravascular Large B-Cell Lymphoma

Intravascular large B-cell lymphoma (IVLBCL) is a rare type of extranodal large B-cell lymphoma characterized by the selective growth of malignant B-cells within the lumina of vessels [2], without nodal or extranodal involvement, occurring in adults. By the time of presentation most patients have advanced, disseminated disease, and the clinical diagnosis is often made at autopsy [208].

The fourth edition of the WHO Classification established two major clinical variants of IVLBCL, the classic and hemophagocytic syndrome-associated forms, but Narittee and colleagues proposed a cutaneous form [131]. Patients with the classic form (Western countries) present symptoms related to the main organ involved, particularly the skin and the central nervous system. The hemophagocytic syndrome-associated form (Asian variant) is characterized by multiorgan failure, pancytopenias, and hepatosplenomegaly. The cutaneous form is confined to the skin, and it has been identified in Western females and is associated with a better prognosis [2,131]. B symptoms, especially fever (45%), are very common, as well as laboratory abnormalities like high serum lactate dehydrogenase (LDH) and soluble IL-2 receptor (sIL-2R) levels [2,131].

Microscopically, the neoplastic lymphoid cells are medium to large size, with prominent nucleoli, scant cytoplasm, and frequent mitotic figures [2,131,208]. Tumor cells express mature B-cell antigens, and about 30% of cases are CD5 positive [2,131,208]. CD10 (10%), BCL-2 (90%), C-MYC (70%), IRF4/MUM-1 (75%), and BCL-6 (25–60%) can also be positive. Ki-67 is usually high, from 60% to 100%. PD-L1 can be positive in 44–50% of cases in any clinical variant [131].

WES results recently showed an ABC-DLBC-like molecular signature, with higher frequencies of mutations in *MYD88*, *CD79B*, *SETD1B*, and *HLA-B* compared to nodal DLBCLs, as well as PD-L1/PD-L2 rearrangements and PD-L1 overexpression [209]. Immunoglobulin genes are clonally rearranged, and karyotypic abnormalities have been described [2] in chromosome 1 (72%), 3, 6q, 14, and 18, as have mutations in *PIM1* (67%), *MYD88* (44–59%), *CD79B* (26–67%), *PRDM1* (44%), *BTG2* (22%), and *EZH2* (4%) [2,131,208]. A defect in homing receptors on the neoplastic cells, such as the lack of CD29 (integrin beta-1) and CD54 (ICAM1) adhesion beta-molecules, might explain the intravascular location [2,131].

## 7. Conclusions

New advances in molecular technologies have yielded a tremendous amount of information about lymphoma biology, leading to a refined classification of lymphomas, which is reflected in the revised fourth edition of the WHO classification. Lymphoma diagnosis currently integrates clinical information, morphology, immunophenotypic, and genetic data. This review focuses on specific variants of DLBCL with unique features and for which recognition is important for diagnosis and therapeutic management, as well as on the large category of DLBCL-NOS that represents 80% of all DLBCL cases.

## Figures and Tables

**Table 1:** Diffuse large-B cell lymphomas reviewed in this paper.

Diffuse Large B-Cell Lymphoma, Nos
High Grade B-Cell Lymphoma
High grade B-cell lymphoma with *MYC* and *BCL2* and/or *BCL6* rearrangement
High grade B-cell lymphoma, NOS
Burkitt lymphoma
Burkitt-like lymphoma with 11q aberration
T-Cell/Histiocyte-Rich Large B-Cell Lymphoma
Diffuse Large B-Cell Lymphoma at Specific Sites
Large B-cell lymphoma with *IRF4* rearrangement (LBCL-IRF4)
Primary mediastinal (thymic) large B-cell lymphoma (PMBL)
Primary cutaneous diffuse large B-cell lymphoma, leg type (PCDLBCL-LT)
Primary diffuse large B-cell lymphoma of the central nervous system (CNS-DLBCL)
Large B-Cell Lymphomas with Plasmacytic Differentiation
ALK-positive large B-cell lymphoma (ALK + DLBCL)
Intravascular large B-cell lymphoma (IVLBCL)

**Table 2:** Main molecular alterations characterizing diffuse large-B-cell lymphoma variants.

Entity/Category	Molecular Alterations		
	Translocations	CNV	Mutations
DLBCL-NOS	*MYC* translocations (10–15%) with *IGH*, I*GL*, I*GK*, *PAX5*, *BCL6*, *BCL11A*, *IKZF1* (*IKAROS*), and *BTG1*.	*PD-L1*/*L2* amplification (20–25%)	*MYD88L265P*, *CD79B*, *NOTCH1*, *EZH2*, *SGK1*, *TET2*, *TP53*
DLBCL-NOS, ABC-subtype	t(14; 3) (q27; q32)/*IGH*-*BCL6* (30%)		*MYD88 (20)*, *CD79A*/*B* (20%), *CARD11* (10%), *MALT1*, *BCL10*, *TNFAIP3*, *PRDM1*/*BLIMP1 (30%)*, *MYD88*, *PIM1*, *NOTCH2*
DLBCL-NOS, GCB-subtype	t(14; 18) (q21; q32)/*IGH*-*BCL2*		*EZH2 (20%)*, *EP300*, *CREBBP*, *KMT2D*, *GNA13*, *GNAI2*, *SIPR2*, *BRAF*, *STAT3*, *RHOA*, *SGK1*, *CARD11*, *NFKBIE*, *NFKBIA*, *CD83*, *CD58*, *CD70*, *BCL2*, *MEF2B*, *IRF8*, *TNFSF14*, *HCNV1*, *PTEN*, *TP53*, *CDKN2A*
HGBL-DH (MYC/BCL2)	*MYC* translocations with 14q32 (*IGH;* 39%), 22q11 (*IGL*; 6%), 2p12 (*IGK*, 7%), 1p36, 3p25, 3q27(*BCL6*), 4p13, 5q13, 9p13 (*PAX5*), 12p11, and 13q31.,	Gains of chromosomes 7, 8, 11, 12, 18, 20 and X, and losses of 3q, 6q, and 15q26	*CREBBP (80%)*, *BCL2 (60%)*, *KMT2D (60%)*, *MYC (45%)*, *EZH2 (40%)*, *IGLL5 (45%)*, *FOX01 (30%)*, *ID3*, *CCND3*, *TCF3*, *EP300*, *MEF2B*, *SGK1*, *SOCS1*, *CCND3*, *TP53*.
HGBL-DH (MYC/BCL6)	*MYC* translocations with 14q32 (*IGH*), 22q11 (*IGL*), 2p12 (*IGK*), 1p36, 3p25, 3q27(*BCL6*), 4p13, 5q13, 9p13 (*PAX5*), 12p11, and 13q31		
HGBL-TH	*MYC* translocations with 14q32 (*IGH*), 22q11 (IGL), 2p12 (IGK), 1p36, 3p25, 3q27(BCL6), 4p13, 5q13, 9p13 (PAX5), 12p11, and 13q31		*TP53*, *BCL2*, *CCND3*, *CREBBP*, *EZH2*, *ID3*, *KMT2D*, *MYC*, *FOXO1*, and *SOCS1*.
HGBCL, NOS		Amplifications of *MYC*, *BCL2*, or *BCL6*.	Isolated MYC (40%), BCL2, or BCL6 breakpoints. Simultaneous rearrangement of BCL6 and BCL2.
BL	t(8; 14) (q24; q32) IGH/MYC (80%), t(2; 8)(q12;24) IGK/MYC (15%) and t(8; 22) (q24;11) IGL/MYC (5%)	1q gain	*TCF3* or its negative regulator *ID3* (40–70%), *CCDN3* (38%), *GNA13*, *RET*, *PIK3R1*, *DDX3X*, *FBXO11*, *SWI*/*SNF* genes, *ARID1A*, *SMARCA4*, *BCL7A*, *BCL6*, *DNMT1*, *SNTB2*, *CTCF*, *IGLL5*, *BACH2*, *TP53* (40%)
BLL, 11Q		Gains of 12q12-q21.1, losses of 6q12.1-q21.1	Mutations involving *BTG2*, *DDX3X*, *ETS1*, *EP300*, and *GNA13*
THRLBCL	*PAX5*/*IGH* rearrangements	Gains of 2p16.1 and PD-L1/PD-L2 (64%). Losses of 2p11.2, and 9p11.2	*JUNB*, *DUSP2*, *SGK1*, *SOC1*
LBCL-IRF4	*IRF4*, *BCL6* rearrangements *IRF4*/*IGH* fusions	Gains of 7q32.1-qter, 11q22.3-qter, and Xq28 Losses of 6q13–16.1, 15q14–22.3, 17p, and *TP53*	*CARD11*, *CD79B*, and *MYD8*
PMBL		Amplification of *REL (75%)* and *BCL11A (50%)* Deletions of *NFKBIE* (20%) 9p24.1 (70%)	*TNFAIP3* (60%), *GNA13* (50%), *BCL6* (50%), *XPO1*(35%), *STAT6* (72%), *SOCS1* (45%), *PTPN1* (25%), *IL4R* (24,2%), *CIITA*, *CD58*, *B2*, *PD-L1*, and *PD-L2*
PCDLBCL-LT	*Translocations involving BCL6*, *MYC*, and *IGH* *PD-L1*/*PD-L2* (40%), *IGH-IRF8*	Amplification of *BCL2*, 18q21 Loss of *CDKN2A* and *CDKN2B*, 6q deletions (loss of *BLIMP1*)	*MYD88* (75%), *TNFAIP3* (40%), *CD79A*, *CD79B*, *CARD11*, *NFKBIE*
CNS-DLBCL	*MYC*, *BCL2*, and *BCL6* translocations	Gains 18q21.33–23, 10q23.21 and 9p24.1. Deletions of 6p21, 6q and 9q21.3	*PIM1* (77%), *MYD88* (64%), *CD79B* (59%), *CDKN2A*, *IRF4*, *CARD11*, *CD79A*, *ITPKB*, *KMT2D*, *CREBBP*, *MEF2B*, *CCND1*, *SOCS1*, *STAT6*, *STAT3*, *CD58*, *CIITA*, *GNA13*, *MYC*, *RHO*, *BLC2*, *PTEN*, and *SMO*
ALK+ DLBCL	t(2; 17) (p23; q23) Clathrin-*ALK* (85–90%) t(2; 5) (p23; q35) NPM-ALK, SEC31A-ALK, SQSTM1-ALK, RANBP2-ALK, IGL-ALK, and CLTC-ALK		Insertion of the ALK gene into 4q22–24
IVLBCL	PD-L1/PD-L2 rearrangements		*PIM1* (67%), *MYD88* (44–59%), *CD79B* (26–67%), *PRDM1* (44%), *BTG2* (22%) and *EZH2* (4%).

DLBCL-NOS: Diffuse large B-cell lymphoma, not otherwise specified; DLBCL-NOS, ABC-subtype; Diffuse large B-cell lymphoma, not otherwise specified, activated B-cell phenotype; DLBCL-NOS, GCB-subtype: Diffuse large B cell lymphoma, not otherwise specified, germinal-center B-cell phenotype; HGBL-DH (MYC/BCL2): High grade B-cell lymphoma with *MYC* and *BCL2* rearrangement; HGBL-DH (MYC/BCL6): High grade B-cell lymphoma with *MYC* and *BCL6* rearrangement; HGBL-TH: triple-hit high-grade large B-cell lymphoma; HGBL, NOS: High grade B-cell lymphoma, not-otherwise specified; BL: Burkitt Lymphoma; BL-11QBurkitt-like lymphoma with 11q aberration; THRLBCL: T-cell/Histiocyte-Rich Large B-cell Lymphoma; LBCL-IRF4: Large B-cell lymphoma with *IRF4* rearrangement; PMBL: Primary mediastinal (thymic) large B-cell lymphoma; PCDLBCL-LT: Primary cutaneous diffuse large B-cell lymphoma, leg type; CNS-DLBCL: Primary diffuse large B-cell lymphoma of the central nervous; ALK + DLBCL: ALK-positive large B-cell lymphoma; IVLBCL: Intravascular large B-cell lymphoma.
